# Alpha- and beta- adrenergic receptors regulate inflammatory responses to acute and chronic sleep fragmentation in mice

**DOI:** 10.7717/peerj.11616

**Published:** 2021-06-24

**Authors:** Nicholas D. Wheeler, David C. Ensminger, Megan M. Rowe, Zachary S. Wriedt, Noah T. Ashley

**Affiliations:** 1 Department of Biology, Western Kentucky University, Bowling Green, KY, United States of America; 2 College of Veterinary Medicine, Mississippi State University, Starkville, MS, United States of America; 3 Department of Integrative Biology, University of California, Berkeley, Berkeley, CA, United States of America

**Keywords:** Sleep Fragmentation, Sympathetic Nervous System, Inflammation, Adrenergic Receptor, Cytokines, Obstructive sleep apnea

## Abstract

Sleep is a recuperative process, and its dysregulation has cognitive, metabolic, and immunological effects that are largely deleterious to human health. Epidemiological and empirical studies have suggested that sleep fragmentation (SF) as result of obstructive sleep apnea (OSA) and other sleep abnormalities leads to pronounced inflammatory responses, which are influenced by the sympathetic nervous system (SNS). However, the underlying molecular mechanisms contributing to SNS regulation of SF-induced inflammation are not fully understood. To assess the effects of the SNS upon inflammatory responses to SF, C57BL/6j female mice were placed in automated SF chambers with horizontally moving bars across the bottom of each cage at specified intervals to disrupt sleep. Mice were first subjected to either control (no bar movement), acute sleep fragmentation (ASF), or chronic sleep fragmentation (CSF) on a 12:12-h light/dark schedule. ASF involved a bar sweep every 120 s for 24 h, whereas CSF involved a bar sweep every 120 s for 12 h (during 12 L; resting period) over a period of 4 weeks. After exposure to these conditions, mice received an intraperitoneal injection of either phentolamine (5 mg/kg BW; an α-adrenergic receptor blocker), propranolol (5 mg/kg BW; a β-adrenergic receptor blocker), or vehicle (saline). Serum corticosterone concentration, brain and peripheral cytokine (IL1β, TNFα, and TGFβ) mRNA expression, and body mass were assessed. ASF and CSF significantly elevated serum corticosterone concentrations as well as cytokine mRNA expression levels compared with controls, and mice subjected to CSF had decreased body mass relative to controls. Mice subjected to CSF and treated with phentolamine or propranolol had a greater propensity for a decrease in cytokine gene expression compared with ASF, but effects were tissue-specific. Taken together, these results suggest that both α- and β-adrenergic receptors contribute to the SNS mediation of inflammatory responses, and adrenergic antagonists may effectively mitigate tissue-specific SF-mediated inflammation.

## Introduction

Sleep is restorative, and its dysregulation can have lead to cognitive, metabolic, and immunological consequences that can have deleterious effects upon human health. Obstructive sleep apnea (OSA) is characterized by repeated episodes of airway obstruction, intermittent oxygen saturation, and sleep disruption, and this condition had become more prevalent due to the obesity epidemic (Peppard et al., 2000; Young et al., 2002). It is well known that OSA, as well as sleep disruptions in general, lead to inflammatory responses in the brain and periphery (Frey, Fleshner & Wright, 2007; Wisor, Schmidt & Clegern, 2011). Dysregulated sleep and OSA are associated with increased circulating concentrations of proinflammatory cytokines, including interleukin (IL)-1, IL-6, and tumor necrosis factor (TNF)-α (Faraut et al., 2012), which are important molecular signatures of a pro-inflammatory response (Ashley, Weil & Nelson, 2012). The progressive build-up of inflammatory insults can promote a systemic chronic inflammatory state that can lead to cardiovascular and metabolic disease, as well as cancer and neurogenerative disorders (Straub & Schradin, 2016; Furman et al., 2019)

It is well known that hormonal responses regulate inflammatory responses (Silverman & Sternberg, 2012; Cain & Cidlowski, 2017); however, there are few empirical studies investigating these responses within the context of sleep loss (see Mishra et al., 2020). The activation of the hypothalamic-pituitary-adrenal (HPA) axis and sympathetic nervous system (SNS) are commonly identified as physiological stress responses (Suchecki et al., 1998; Meerlo, Sgoifo & Suchecki, 2008), and can be triggered by sleep restriction or deprivation. Sleep curtailment can increase SNS activity and subsequently an elevation of norepinephrine (NE) released through noradrenergic neurons and epinephrine and NE from the adrenal medullae (Dimsdale et al., 1995; Tiemeier et al., 2002; Mishra et al., 2020). Glucocorticoids are released from adrenal cortices via HPA activation (Suchecki et al., 1998; Meerlo, Sgoifo & Suchecki, 2008) several minutes after SNS stimulation. While effects of acute sleep loss on SNS and HPA activity can be considered mild or adaptive, chronic sleep loss caused by OSA, shift work, and modern lifestyles can contribute to more deleterious effects such as cardiovascular and metabolic disease, obesity and neurological disorders (Schwartz et al., 1999; Mavanji et al., 2012). Our laboratory previously showed that SF-induced increases in cytokine gene expression and serum proteins were mitigated by chemical sympathectomy, confirming that inhibition of the SNS reduces inflammatory responses from acute and chronic SF (Mishra et al., 2020). However, it is unknown how NE is acting on these target tissues to regulate inflammation.

The aim of this study was to assess the role of α- and β- adrenergic receptors in modulating inflammatory responses to acute and chronic SF. To test the effect of the SNS on inflammatory responses to SF, female C57BL/6 mice were subjected to ASF, CSF, or control (CON) conditions and injected with either an α-adrenergic receptor antagonist, phentolamine (5 mg/kg BW), a β -adrenergic receptor antagonist, propranolol (5 mg/kg BW), or vehicle (saline). Propranolol is a non-selective competitive β-adrenergic receptor antagonist that also crosses the blood-brain barrier. It blocks the action of catecholamines from binding to both β_1_ and β_2_ adrenergic receptors. Phentolamine is a non-selective α-adrenergic receptor antagonist that has been commonly used to treat hypertension by acting on blood vessels to induce dilation. It competitively blocks both α-1 and α-2 receptors and can cross the blood-brain-barrier (Richards, Woodings & Prichard, 1978; Auer, Trummer & Johansson, 1981; Limberger et al., 1989; Antunes-Rodrigues et al., 1993). Alpha-1 receptors are typically found in vascular smooth muscle while α-2 receptors are detected in the brain and periphery and are thought to modulate sympathetic outflow in the brainstem (Reid, 1986). Brain and peripheral tissues will be assessed for cytokine gene expression, specifically the hypothalamus, hippocampus, and prefrontal cortex (for brain) and spleen, white adipose tissue, liver, and heart (for periphery). These regions were selected based upon past studies that have reported an inflammatory response in those specific tissues from sleep fragmentation (Dumaine & Ashley, 2015; Mishra et al., 2020).

Sleep loss stimulates nerve fibers from the SNS to release the neurotransmitter norepinephrine and bind to leukocyte adrenergic receptors (Irwin & Opp, 2017), which leads to expression of pro-inflammatory cytokines. In the immune system, myeloid cells typically express α- and β-adrenergic receptors, whereas lymphocytes largely express β-adrenergic receptors (Fuchs, Albright & Albright, 1988). As the majority of immune cells in the periphery predominantly express β-adrenergic receptors (Liu & Hong, 2003; Kolmus, Tavernier & Gerlo, 2015), we hypothesized that β-adrenergic receptor blockade would reduce SF-induced inflammatory responses more than α-adrenergic receptor blockade.

## Materials & Methods

### Animals

The mice used for this study were bred from the mouse colony housed in the mouse colony room at Western Kentucky University. Female C57BL/6j mice (n = 120) were housed in our colony room (12:12-h light-dark cycle, lights on at 0800, 21 °C ±1 °C) at Western Kentucky University. After weaning at 21 days of age, female mice were separated into polypropylene cages with same-sex littermates and provided with corncob bedding, and food and water *ad libitum*. Female mice were used in consideration of the NIH Notice Number: NOT-OD-15-102, which highlights the over-reliance on male animals and cells in basic and clinical research and requests for more studies to consider using female organisms. Female mice >8 weeks of age were selected for experiments and placed in automated sleep fragmentation chambers with chambers housing no more than 5 mice (Lafayette Instrument Company; Lafayette, IN; model 80390). Each cage was provided corncob bedding, and food as well as water were *ad libitum*. Mice were tagged with numbered ear tags and then acclimated to the automated sleep fragmentation chamber for 72 h prior to initiating SF experiments. Mice were used as a model for this study due to their anatomical, physiological, and genetic similarity to humans. This study was conducted under the approval of the Institutional Animal Care and Use Committee at Western Kentucky University (#19-14), and the procedures followed the National Institutes of Health’s “Guide for the Use and Care of Laboratory Animals” and international ethics standards. Mice were to be euthanized if injured or in physiological distress; however, this was not necessary for our study.

### Experiment 1: Acute Sleep Fragmentation (ASF)

Sample sizes were aimed at having at least 6 mice per group and groups were randomized by weight. The order of treatments and location of mouse cages were randomly designated and was only aware to the first author. Fifty-three mice received the following pharmacological treatments at 07:30 (30 min before lights on) using a single intraperitoneal injection: 18 mice were treated with phentolamine (5.0 mg/kg BW; an α-adrenergic receptor blocker), 18 mice were treated with propranolol (5.0 mg/kg BW; a β-adrenergic receptor blocker), and 17 mice were treated with vehicle (0.9% NaCl solution). Dosages of phentolamine and propranolol were based upon previous studies that effectively inhibited signaling of the sympathetic nervous system while under varying types of stressful stimuli (Allison et al., 1969; Hermansen & Hyttel, 1971; Fabian et al., 1973; Hall et al., 1987; Jun et al., 2014). Thirty minutes following injections (08:00, lights on), experimental mice (*n* = 27; *n* = 9 injected with phentolamine, *n* = 9 injected with propranolol, and *n* = 9 injected with vehicle) were subjected to ASF; i.e., the sweeping bar set to move horizontally at an interval of 120 seconds for 24 h. This rate is comparable to sleep disruptions that occur in humans with severe sleep apnea (Ramesh, Kaushal & Gozal, 2009, Goyal & Johnson, 2017). The remaining mice (*n* = 26; *n* = 9 injected with phentolamine, *n* = 9 injected with propranolol, and *n* = 8 injected with vehicle) were not subjected to any bar sweeps (controls), but were still housed in the SF chamber.

### Experiment 2: Chronic Sleep Fragmentation (CSF)

**Figure 1:**
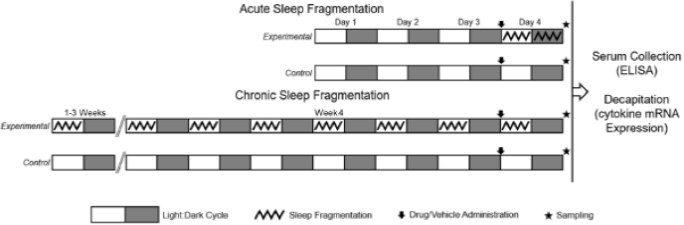
Experimental protocol for ASF and CSF studies. Experimental protocol for the two experiments performed. Experimental mice subjected to sleep fragmentation (SF) in an automated SF chamber for 24 h (experiment 1: Acute SF) or 4 weeks (experiment 2: Chronic SF). An automated horizontally sweeping bar moved across the bottom of the chamber every 2 min to ensure that sleep was regularly disrupted. The control (CON) groups of each experiment were also contained in their own SF chambers, however, the bars inside the chambers remained stationary. Mice were acclimated to the SF chambers for 72 h (days 1–3) prior to the initiation of the experiments. In the Acute SF experiment (ASF), control and experimental mice received an intraperitoneal injection of 0.9% saline (vehicle), phentolamine, or propranolol 30 minutes before initiating their respective sleep treatments. After 24 h of ASF or CON treatment, mice (SF: n = 9/group; CON: n = 10/group) were decapitated for tissue gene expression quantification. In the Chronic SF (CSF) experiment, control and experimental received an intraperitoneal injection of 0.9% saline (vehicle), phentolamine, or propranolol 24 h before the conclusion of the chronic sleep treatments. After 4 weeks of CSF or CON sleep treatment, mice (SF: n = 10/group; CON: n = 10/group) were decapitated for tissue gene expression quantification. All mice were >8 weeks of age, were subjected to 12 h light: 12 h dark cycles with lights on at 8:00am and lights off at 8:00pm, and were provided food and water *ad libitum*.

Sample sizes were aimed at having at least 6 mice per group and groups were randomized by weight. The order of treatments and location of mouse cages were randomly designated, and was only aware to the first author. To induce CSF, experimental mice (*n* = 30) were subjected to a horizontal sweeping bar that moved every 120 s (30 swipes/h) during the light phase (from 8:00 to 20:00) every day for 4 weeks (28 days), while the control mice (*n* = 30) were not subjected to any bar sweeps. On the 27th day of CSF, 24 h prior to tissue collection, experimental mice (*n* = 30; *n* = 10 injected with phentolamine, *n* = 10 injected with propranolol, and *n* = 10 injected with vehicle) and control mice (*n* = 30; *n* = 10 injected with phentolamine, *n* = 10 injected with propranolol, and *n* = 10 injected with vehicle) received their respective injections at 07:30 (a total of 60 mice were used). Mice were weighed on an electronic scale (to the nearest 0.1 g) every week to track changes in body mass in response to CSF. A schematic of the overall experimental design is presented in Fig. 1.

### Sample collection

In both experiments, 24.5 h following drug administration (08:00), mice were deeply anesthetized using isoflurane vapors (<2min) and rapidly decapitated in <3 min of initial handling for tissue gene expression studies and measurement of baseline serum corticosterone concentration. Trunk blood from decapitated mice was collected, kept on ice for < 20 min, and then spun at 3,000× g for 30 min at 4 °C. The serum was collected and stored at −20 °C for later ELISA analyses. For gene expression studies, the brain, extra-oviductal white adipose tissue (EOWAT), heart, liver, and spleen were dissected from mice and stored in RNAlater solution (ThermoFischer Scientific). Brain samples were later dissected and pre-frontal cortexes, hippocampi, and hypothalami were collected, and placed in RNAlater. All tissue samples were stored at 4 °C for no more than 30 days before RNA extraction (see below).

### ELISA

Serum levels of corticosterone (*n* = 6–10/group) were measured as per the manufacturer’s protocols (Catalogue number: ADI-900-097, EnzoLife Sciences; Abcam). Average intra- and inter-assay coefficients of variation were 4.6% and 7.2%, respectfully.

### Real time-PCR

The following protocols were described previously by Mishra et al. (2020). Briefly, RNA was extracted from EOWAT, heart, liver, spleen, and brain tissue using RNeasy mini kits (Qiagen). RNA concentrations were assessed with a NanoDrop 2000 Spectrophotometer (ThermoScientific). Using a high-capacity cDNA reverse transcription kit (Life Technologies, Cat number:1384368813, Total RNA was reverse transcribed into cDNA. The relative cytokine gene expression was determined by using the prepared cDNA as a template and running samples on a ABI 7300 RT-PCR system. Cytokine probes (IL1β: Mm00434228 , TNFα: Mm00443258 , TGFβ: Mm00447500; Applied Biosystems) labelled with fluorescent marker 5-FAM at the 5′ end and quencher MGB at the 3′ end was used for genes of interest. A primer-limited 18S probe (4319413E; Applied Biosystems) was used as the endogenous control. Samples were run in duplicate. Cycle threshold (Ct) was used to calculate the relative expression in mRNA levels of the genes of interest relative to the endogenous control using a standard curve. The relative expression was then converted to fold change. A standard curve was created by injecting a mouse with 100 µL lipopolysaccharide (1 mg/kg BW) to induce a severe pro-inflammatory response, extracting RNA from the liver, and then reverse transcribing the RNA into cDNA. The cDNA was used to create a ten-fold series dilution (1:1, 1:10, 1:100, 1:1000, 1:10000) to generate the standard curve plot points. Outliers were removed based upon a two-sigma analysis.

### Statistical analyses

Data are presented as mean (± SE). Statistical analyses were conducted in R Studio (v.1.1.463, R Development Core Team, Boston, MA) and figures were visualized in GraphPad Prisim 8 (Version 8.4.3 (686)). Animals and data points were excluded from the analysis if the RealTime PCR or ELISA readings were undetermined. A two-way ANOVA assessed the effect of sleep fragmentation (ASF and CSF), the effect of the adrenergic receptor blockers (propranolol and phentolamine), and the interaction effect of ASF and CSF with the pharmacological blocker on cytokine mRNA expression and serum corticosterone concentration. The interaction term was removed from the model if it was nonsignificant to preserve degrees of freedom. Tukey’s HSD test was used for post-hoc analysis. Logarithmic transformation was used to satisfy the requirement of homogeneity of variances. A one-way repeated measures ANOVA assessed the effect of CSF, time (the repeated measure), and their interaction effect upon body mass. Tukey’s HSD test was used for post-hoc analysis. Results are presented as means ± 1 SE, and *p* < 0.05 was considered statistically significant.

## Results

### Acute Sleep Fragmentation (ASF)

#### Serum corticosterone

**Figure 2:**
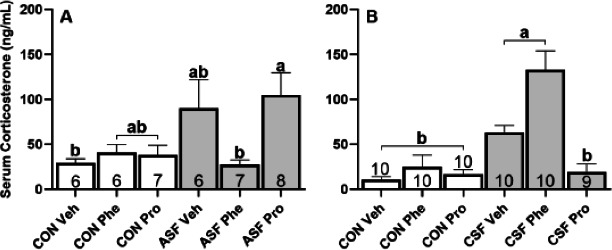
ASF and CSF experiments—serum corticosterone concentration. Effects of acute sleep fragmentation (ASF, Fig. 4A) and chronic sleep fragmentation (CSF, Fig. 4B), adrenergic blockade (phentolamine (Phe) or propranolol (Pro) or vehicle (Veh), and their interaction on serum corticosterone levels. Sample sizes of each treatment group are listed with their respective bar graph and were analyzed using a two-way ANOVA and Tukey’s HSD post hoc tests. Data shown as means 1 SE for each group and differing letters denotes *p* < 0.05.

ASF increased serum corticosterone (Cort) concentration (*F*_1,34_ = 5.66, *p* = 0.003 Fig. 2A) while drug treatment had no effect on serum Cort (*F*_2,34_ = 2.78, *p* = 0.08, Fig. 2A). An interaction between drug and sleep treatments was present (*F*_2,34_ = 4.58, *p* = 0.02, Fig. 2A); ASF Propranolol (Pro) exhibited significantly elevated serum Cort relative to Control (CON) Vehicle (Veh) and ASF Phentolamine (Phe) groups (Tukey’s HSD, *p* < 0.05).

#### Brain response

**Figure 3:**
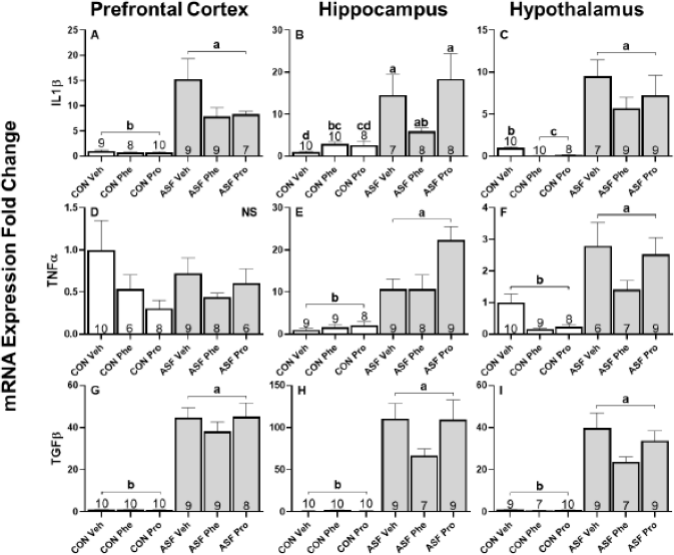
ASF experiment—cytokine mRNA expression in brain. Effects of acute sleep fragmentation (ASF), adrenergic blockade, and their interaction on cytokine (IL1β, TNFα, and TGFβ) mRNA expression in prefrontal cortex (A, D, G), hippocampus (B, E, H), and hypothalamus (C, F, I) of mice injected with a pharmacological adrenergic block (phentolamine (Phe) or propranolol (Pro) or vehicle (Veh) and were either subjected to control (CON) or ASF. Sample sizes of each treatment group are listed with their respective bar graph and were analyzed using a two-way ANOVA and Tukey’s HSD post hoc tests. Data shown as means 1 SE for each group and differing letters denotes *p* < 0.05.

ASF significantly increased gene expression of IL1β (*F*_1,48_ = 161.52, *p* < 0.001, Fig. 3A) and TGFβ (*F*_1,52_ = 1620.92, *p* < 0.001, Fig. 3G), but did not affect the expression of TNFα (*F*_1,43_ = 0.68, *p* = 0.41, Fig. 3D) compared to controls. There was no significant effect of drug treatment upon expression of any of the cytokines (IL1β, *F*_2,48_ = 2.02, *p* = 0.14, Fig. 3A; TNFα, *F*_2,43_ = 2.40, *p* = 0.10, Fig. 3D; TGFβ*, F*_2,52_ = 0.97, *p* = 0.39, Fig. 3G).

ASF significantly increased cytokine gene expression in hippocampus (IL1β: *F*_1,45_ = 55.29, *p* < 0.001, Fig. 3B; TNFα: *F*_1,46_ = 94.89, *p* < 0.001, Fig. 3E; TGFβ: *F*_1,49_ = 1055.80, *p* < 0.001, Fig. 3H) compared to controls. There was a significant effect from drug treatment on TNFα expression (*F*_2,48_ = 4.813, *p* = 0.01, Fig. 3E), but not on IL1 β expression (*F*_2,45_ = 2.42, *p* = 0.10, Fig. 3A) or TGFβ (*F*_2,49_ = 0.62, *p* = 0.54, Fig. 3H). Tukey’s HSD showed that Pro increased TNFα expression levels compared to Phe and Veh. There was an interaction effect between drug and sleep treatments for IL1β expression (*F*_2,45_ = 5.39, *p* = 0.007, Fig. 3B) and TGF β expression (*F*_2,45_ = 5.012, *p* = 0.01, Fig. 3H); however, post-hoc tests revealed only a significant difference between sleep treatments for each gene.

In hypothalamus, ASF significantly increased cytokine gene expression (IL1β: *F*_1,47_ = 275.75, *p* < 0.001, Fig. 3C; TNFα: *F*_1,45_ = 49.54, *p* < 0.001, Fig. 3F; TGFβ: *F*_1,47_ = 1373.84, *p* < 0.001, Fig. 3I). Drug treatment also had an effect on cytokine gene expression (IL1β: *F*_2,47_ = 18.23, *p* < 0.001, Fig. 3C; TNFα: *F*_2,45_ = 6.83, *p* = 0.002, Fig. 3F; TGFβ: *F*_2,47_ = 1373.84, *p* < 0.001, Fig. 3I). Specifically, Pro and Phe decreased IL1β and TGFβ expression relative to Veh while TNFα expression was reduced by just Phe. There was an interaction effect between drug and sleep treatments influencing IL1β expression (*F*_2,47_ = 0.97, *p* < 0.001, Fig. 3C); post-hoc tests revealed that ASF groups had higher expression levels than CON treated groups and that CON Veh had higher expression levels than CON Phe and Pro.

#### Peripheral response

**Figure 4:**
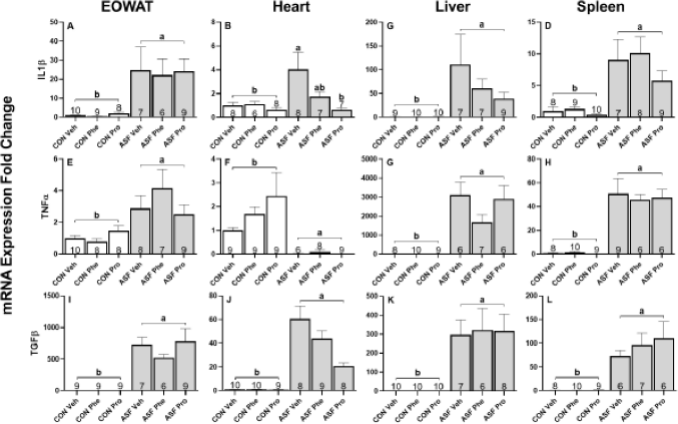
ASF Experiment—cytokine mRNA expression in peripheral tissues. Effects of acute sleep fragmentation (ASF), adrenergic blockade, and their interaction on cytokine (IL1β, TNFα, and TGFβ) mRNA expression levels in EOWAT (A, E, I), heart (B, F, J), liver (C, G, K), and spleen (D, H, L) of mice injected with a pharmacological adrenergic block (phentolamine (Phe) or propranolol (Pro) or vehicle (Veh) and were either subjected to control (CON) or ASF. Sample sizes of each treatment group are listed with their respective bar graph and were analyzed using a two-way ANOVA and Tukey’s HSD post hoc tests. Data shown as means 1 SE for each group and differing letters denotes *p* < 0.05.

ASF significantly increased gene expression of IL1β (*F*_1,45_ = 156.50, *p* < 0.001, Fig. 4A), TNFα (*F*_1,46_ = 24.5, *p* = 23.14, Fig. 4E), and TGFβ (*F*_1,43_ = 1048.37, *p* < 0.001, Fig. 4I) compared to controls in EOWAT. Drug treatment significantly altered the expression of IL1β (*F*_2,45_ = 6.31, *p* = 0.004, Fig. 4A) and TGFβ (*F*_2,43_ = 4.83, *p* = 0.01, Fig. 4I) but not TNF α (*F*_2,46_ = 0.58, *p* = 0.56, Fig. 4E) relative to Veh. Post-hoc Tukey’s test revealed that Phe decreased IL1β expression when compared to Pro whereas Pro increased TGFβ expression compared with Veh.

In cardiac tissue, ASF significantly increased gene expression of IL1β (*F*_1,38_ = 12.70, *p* = 0.001, Fig. 4B) and TGFβ (*F*_1,48_ = 805.94, *p* < 0.001, Fig. 4J) compared with controls, however, TNFα (*F*_1,45_ = 356.66, *p* < 0.001, Fig. 4F) expression levels were significantly lower than that of the controls. There was a drug effect on the expression of IL1β (*F*_2,38_ = 8.42, *p* < 0.001, Fig. 4B) and TGFβ (*F*_2,48_ = 7.22, *p* = 0.002, Fig. 4J). Post-hoc tests showed that Pro suppressed the expression levels of IL1β and TGFβ relative to Veh. There was no effect of the pharmacological blockade on the expression of TNFα (*F*_2,43_ = 0.19, *p* = 0.83, Fig. 4F). There was an interaction effect on IL1β gene expression (*F*_2,38_ = 3.42, *p* = 0.04, Fig. 4B); post-hoc tests revealed that ASF Pro had IL1β expression levels equal to that of CON groups, and that ASF Pro expression was significantly lower than ASF Veh, but not ASF Phe (Fig. 4B).

ASF treatment significantly increased hepatic gene expression of each cytokine assessed (IL1β: *F*_1,48_ = 212.25, *p* < 0.001, Fig. 4C; TNFα: *F*_1,42_ = 2153.85, *p* < 0.001, Fig. 4G; TGFβ: *F*_1,47_ = 947.400, *p* < 0.001, Fig. 4K) relative to controls. There was no significant drug effect on the expression of TGFβ (*F*_2,47_ = 2.78, *p* = 0.07, Fig. 4K), IL1β (*F*_2,48_ = 0.32, *p* = 0.73, Fig. 4C), or TNFα (*F*_2,42_ = 1.86, *p* = 0.17, Fig. 4G).

In spleen, ASF increased cytokine gene expression (IL1β: *F*_1,46_ = 65.294, *p* < 0.001, Fig. 4D; TNFα: *F*_1,44_ = 189.456, *p* < 0.001, Fig. 4H; TGFβ: *F*_1,45_ = 575.832, *p* < 0.001, Fig. 4L) compared with controls. Drug treatment significantly decreased the expression of TNFα (*F*_2,44_ = 5.648, *p* = 0.00656, Fig. 4H) and TGF β (*F*_2,45_ = 4.936, *p* < 0.0115, Fig. 4L) with Pro and Phe, respectively, relative to Veh injection. The effect of drug treatment on IL1β expression was not statistically significant (*F*_2,46_ = 3.188, *p* = 0.0505, Fig. 4D). Table 1 provides a summary of results for ASF effects, drug effects, and interaction effects on cytokine gene expression in brain and peripheral tissues.

**Table 1:** Summary of ASF results for cytokine gene expression in brain and peripheral tissues.

Tissue	**Sleep fragmentation**	**Phentolamine**	**Propranolol**	**Interaction**
Prefrontal cortex	Increase: IL1β & TGFβ	No Effect	No Effect	No Effect
Hippocampus	Increase: IL1β, TNFα, & TGF β	No Effect	Increase: TNFα	No Effect
Hypothalamus	Increase: IL1β, TNFα, & TGFβ	Decrease: IL1β, TNFα, & TGFβ	Decrease: IL1β & TGFβ	Decrease: IL1 β (CON Phe & CON Pro)
EOWAT	Increase: IL1β, TNFα, & TGFβ	Decrease: IL1β	Increase: TGFβ	No Effect
Heart	Increase: IL1β & TGFβ Decrease: TNFα	No Effect	Decrease: IL1β & TGFβ	Decrease: IL1β (ASF Pro) & TGFβ (ASF Pro)
Liver	Increase: IL1β, TNFα, & TGFβ	No Effect	No Effect	No Effect
Spleen	Increase: IL1β, TNFα, & TGFβ	Decrease: TGFβ	Decrease: TNFα	No Effect

**Notes.**

ASF Experiment—Summary of ASF effects, drug effects, and interaction effects on cytokinegene expression in brain and peripheral tissues.

### Chronic Sleep Fragmentation (CSF)

#### Serum corticosterone

CSF increased serum Cort concentration (*F*_1,53_ = 14.11, *p* < 0.001, Fig. 2B) relative to controls, while drug treatment also altered serum corticosterone concentrations (*F*_2,53_ = 8.20, *p* < 0.001, Fig. 2B). The interaction effect between drug and sleep treatments also had a significant effect on corticosterone (*F*_2,34_ = 4.58, *p* = 0.02, Fig. 2B); post-hoc tests revealed that corticosterone concentrations of CSF Pro was not significantly different from controls while CSF Phe and CSF Veh had significantly higher circulating concentrations than the controls.

#### Brain response

**Figure 5:**
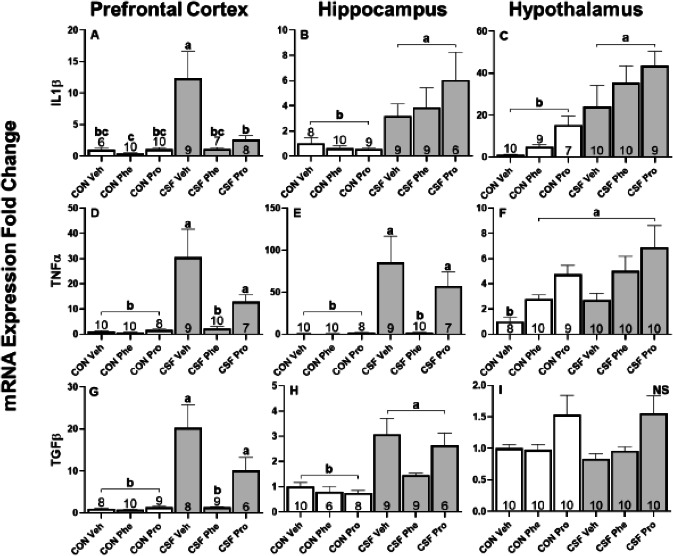
CSF experiment—cytokine mRNA expression in brain. Effects of chronic sleep fragmentation (CSF), adrenergic blockade, and their interaction on cytokine (IL1β, TNFα, and TGFβ) mRNA expression levels in prefrontal cortex (A, D, G), hippocampus (B, E, H), and hypothalamus (C, F, I) of mice injected with a pharmacological adrenergic block (Phentolamine (Phe) or Propranolol (Pro) or vehicle (Veh) and were either subjected to control (CON) or (CSF). Sample sizes of each treatment group are listed with their respective bar graph and were analyzed using a two-way ANOVA and Tukey’s HSD post hoc tests. Data shown as means 1 SE for each group and differing letters denotes *p* < 0.05.

In prefrontal cortex, CSF significantly increased cytokine gene expression (IL1β: *F*_1,44_ = 30.70, *p* < 0.001, Fig. 5A; TNFα: *F*_1,49_ = 39.39, *p* < 0.001, Fig. 5D; TGF β: *F*_1,44_ = 66.60, *p* < 0.001, Fig. 5G) relative to controls. There was also a significant effect of drug treatment on cytokine gene expression (IL1 β, *F*_2,44_ = 30.70, *p* < 0.001, Fig. 5A; TNFα, *F*_2,49_ = 7.34, = 0.002, Fig. 5D; TGFβ*, F*_2,44_ = 18.05, *p* < 0.001, Fig. 5G). Furthermore, an interaction effect between sleep and drug treatments altered cytokine gene expression (IL1β, *F*_2,44_ = 3.51, *p* = 0.04, Fig. 5A; TNF α, *F*_2,49_ = 4.41, *p* = 0.02, Fig. 5D; TGFβ*, F*_2,44_ = 11.57, *p* < 0.001, Fig. 5G). Post-hoc tests showed that Pro and Phe groups significantly decreased IL1 β expression levels compared to Veh among CSF mice. In addition, CSF Phe treatment significantly decreased TNF α and TGFβ expression levels relative to CSF Phe and CSF Veh mice.

CSF significantly increased the gene expression of each cytokine in the hippocampus (IL1β: *F*_1,47_ = 40.94, *p* < 0.001, Fig. 5B; TNFα: *F*_1,49_ = 56.79, *p* < 0.001, Fig. 5E; TGF β: *F*_1,42_ = 58.06, *p* < 0.001, Fig. 5H) compared with controls. Drug treatment had a significant effect on TNFα expression (*F*_2,49_ = 13.97, *p* < 0.001, Fig. 5E), a trending effect on TGFβ (*F*_2,49_ = 0.62, *p* = 0.54, Fig. 5H), and no effect on IL1β (*F*_2,47_ = 0.33, *p* = 0.72, Fig. 5B). Tukey’s HSD showed Phe and Pro- treated groups had lower TNFα expression levels than Veh, while there were no significant differences in drug treatments for TGF β. There was an interaction effect between drug and sleep treatments in TNFα expression (*F*_2,49_ = 9.00, *p* = 0.005, Fig. 5E); post-hoc tests revealed that TNFα expression levels in CSF Phe were significantly less than CSF Pro and Veh, and not significantly different from controls.

In hypothalamus, CSF had an effect on gene expression of IL1 β (*F*_1,50_ = 47.92, *p* < 0.001, Fig. 5C) and TNFα (*F*_1,51_ = 11.74, *p* = 0.001, Fig. 5F) but not on TGF β (*F*_1,47_ = 0.21, *p* = 0.65, Fig. 5I) relative to controls. Drug treatment, however, altered the gene expression of each cytokine assessed (IL1β: *F*_2,50_ = 22.04, *p* < 0.001, Fig. 5C; TNF α: *F*_2,51_ = 16.38, *p* < 0.001, Fig. 5F; TGFβ: *F*_2,47_ = 1373.84, *p* < 0.001, Fig. 5I). Post-hoc tests showed Phe and Pro treated groups had higher IL1β and TNFα expression levels than Veh, and that TGFβ was higher in Pro than in Phe or Veh. There was an interaction effect between drug and sleep treatments influencing TNFα expression (*F*_2,51_ = 3.75, *p* = 0.03, Fig. 5F); however, post-hoc tests revealed that only CON Veh was statistically different from all other treatment groups.

#### Peripheral response

**Figure 6:**
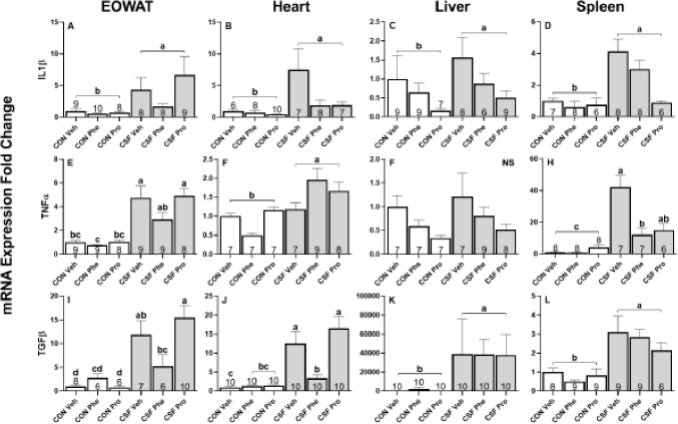
CSF experiment—cytokine mRNA expression in peripheral tissues. Effects of chronic sleep fragmentation (CSF), adrenergic blockade, and their interaction on cytokine (IL1β, TNFα, and TGF*β*) mRNA expression levels in EOWAT (A, E, I), heart (B, F, J), liver (C, F, K), and spleen (D, H, L) of mice injected with a pharmacological adrenergic block (phentolamine (Phe) or propranolol (Pro) or vehicle (Veh) and were either subjected to control (CON) or CSF. Sample sizes of each treatment group are listed with their respective bar graph and were analyzed using a two-way ANOVA and Tukey’s HSD post hoc tests. Data shown as means 1 SE for each group and differing letters denotes *p* < 0.05.

In adipose tissue, CSF significantly increased cytokine gene expression (IL1β: *F*_1,46_ = 40.03, *p* < 0.001, Fig. 6A; TNFα: *F*_1,49_ = 102.07, *p* < 0.001, Fig. 6E; TGF β: *F*_1,44_ = 79.15, *p* < 0.001, Fig. 6I) compared with controls. Drug treatment had a significant effect on the expression of IL1β (*F*_2,45_ = 6.31, *p* = 0.004, Fig. 6A) and TNF α (*F*_2,49_ = 3.38, *p* = 0.04, Fig. 6E), but no effect upon TGFβ. Post-hoc Tukey’s revealed that Phe resulted in a lower IL1β and TNF α expression than when treated with Pro. A significant interaction effect in TGFβ expression (*F*_1,37_ = 10.00, *p* < 0.001, Fig. 6I) was present, and post-hoc tests show CSF Phe had lower expression levels than CSF Pro, which was not significantly different from CON Phe.

In cardiac tissue, CSF significantly increased cytokine gene expression (IL1β: *F*_1,42_ = 27.75, *p* < 0.001, Fig. 6B; TNFα: *F*_1,41_ = 27.47, *p* < 0.001, Fig. 6F; TGFβ: *F*_1,47_ = 92.81, *p* < 0.001, Fig. 6B, 6J) compared with controls. Drug treatment had a significant effect on the expression of each cytokine assessed (IL1 β: *F*_2,45_ = 8.09, *p* = 0.001, Fig. 6B; TNFα: *F*_2,41_ = 12.21, *p* < 0.001, Fig. 6F; TGFβ: *F*_2,37_ = 7.49, *p* = 0.002, Fig. 6J). Post-hoc tests showed Phe and Pro reduced expression of IL1 β, and Phe reduced TNFα expression compared with Pro and Veh. There was an interaction between sleep and drug treatments on TGFβ expression (*F*_2,47_ = 8.16, *p* < 0.001, Fig. 6J) and post-hoc tests indicate that CSF Veh and CSF Pro exhibited high TGFβ expression levels while CSF Phe had expression levels equal to CON Phe and Pro.

In liver, CSF treatment significantly increased the gene expression of IL1β (*F*_1,44_ = 5.72, *p* = 0.021, Fig. 6C) and TGFβ (*F*_1,49_ = 101.72, *p* < 0.001, Fig. 6K), but not TNFα (*F*_1,44_ = 0.02, *p* = 0.89, Fig. 6G), relative to controls. There was no significant drug effect on the expression of TGFβ (*F*_2,49_ = 2.60, *p* = 0.08, Fig. 6K), IL1β (*F*_2,44_ = 1.12, *p* = 0.34, Fig. 6C) or TNF α (*F*_2,44_ = 1.46, *p* = 0.24, Fig. 6G).

In spleen, CSF increased cytokine gene expression (IL1β: *F*_1,39_ = 22.94, *p* < 0.001, Fig. 6D TNFα: *F*_1,38_ = 129.49, *p* < 0.001, Fig. 6H; TGFβ: *F*_1,45_ = 44.17, *p* < 0.001, Fig. 6L) compared with controls. Drug treatment significantly altered the expression of IL1β (*F*_2,39_ = 4.65, *p* = 0.02, Fig. 6D) and TNFα (*F*_2,38_ = 4.13, *p* = 0.02, Fig. 6H) but had no effect on TGFβ (*F*_2,45_ = 1.45, *p* = 0.25, Fig. 6L). Post-hoc test revealed that Pro reduced IL1β expression compared with Phe or Veh, and Phe reduced TNF α expression compared to Pro and Veh. There was an interaction effect between sleep treatment and drug treatment on TNFα expression (*F*_2,38_ = 5.73, *p* = 0.007, Fig. 6H), and post-hoc tests revealed that expression levels in CSF Phe were significantly less than CSF Pro or Veh. Table 2 provides a summary of results for CSF effects, drug effects, and interaction effects on cytokine gene expression in brain and peripheral tissues.

**Table 2:** Summary of CSF results for cytokine gene expression in brain and peripheral tissues.

Tissue	**Sleep fragmentation**	**Phentolamine**	**Propranolol**	**Interaction**
Prefrontal cortex	Increase: IL1β, TNFα, & TGFβ	Decrease: IL1β, TNFα, & TGFβ	Decrease: IL1β	Decrease: IL1β (CSF Phe & Pro), TNFα (CSF Pro), & TGFβ (CSF Phe)
Hippocampus	Increase: IL1β, TNFα, & TGFβ	No Effect	Decrease: TNFα	Decrease: TNFα (CSF Phe)
Hypothalamus	Increase: IL1β & TNFα	Increase: IL1β & TNFα	Increase: IL1β, TNFα, & TGFβ	No Effect
EOWAT	Increase: IL1β, TNFα, & TGFβ	Decrease: IL1β & TNFα	No Effect	Decrease: TGFβ (CSF Phe)
Heart	Increase: IL1β, TNFα, & TGFβ	Decrease: IL1β, TNFα, & TGFβ	Decrease: IL1β	Decrease: TGFβ (CSF Phe)
Liver	Increase: IL1β, TNFα, & TGFβ	No Effect	No Effect	No Effect
Spleen	Increase: IL1β, TNFα, & TGFβ	Decrease: TNFα	Decrease: IL1β	Decrease: TNFα (CSF Phe)

**Notes.**

ASF Experiment—Summary of ASF effects, drug effects, and interaction effects on cytokinegene expression in brain and peripheral tissues.

### Body mass

**Figure 7:**
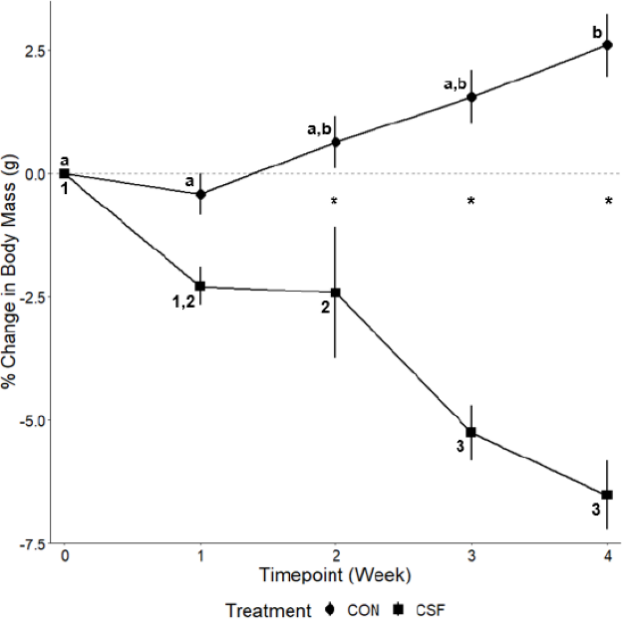
CSF experiment—percentage change in body mass. Effects of chronic sleep fragmentation (CSF) on weekly body mass. Timepoint “Week 0” represents the start of the experiment. Sample sizes are CON n = 60 and CSF = 60, and were analyzed using a repeated-measures ANOVA. Data are shown as means 1 SE for each group and asterisks (*) denotes a significant difference between treatments at each timepoint, letters denote differences within CON, and numbers denote difference within CSF (*p* < 0.05).

There was an effect of sleep treatment (*F*_1,51.481_ = 52.32, *p* < 0.001, Fig. 7), time (*F*_4,246.029_ = 4.47, *p* = 0.02, Fig. 7), and an interaction effect between CSF and time (*F*_4,246.029_ = 24.37, *p* < 0.001, Fig. 7) on body mass. Post-hoc tests revealed that the percent change in body mass for control (CON) and chronic sleep-fragmented (CSF) mice diverged at Week 2 and continued to do so at Week 3 and Week 4. Control mice gained body mass over time whereas CSF mice lost body mass (Fig. 7).

The raw data for this study have been provided as supplemental data files through *Peer-J*.

## Discussion

Our results indicate that adrenergic receptor antagonists, phentolamine and propranolol, had varied effects upon the inflammatory phenotype, and that these effects were dependent upon acute versus chronic sleep fragmentation and tissue type. These results are consistent with a previous study from our lab showing that chemical denervation of sympathetic nerve terminals reduces inflammatory responses in peripheral tissues of female mice (Mishra et al., 2020). This study also confirms findings from previous studies (Dumaine & Ashley, 2015; Mishra et al., 2020) that acute and chronic SF lead to elevated pro-inflammatory gene expression in peripheral and brain tissues. Additionally, it was observed that CSF was a more potent inducer of inflammation than ASF, as also seen in previous research (Mullington et al., 2010; Mishra et al., 2020). However, in some tissues, opposing effects were observed where pharmacological blockade actually increased pro-inflammatory gene expression (e.g., increased TNFα expression in hippocampus from propranolol treatment in the ASF experiment, increased IL1β and TNFα in hypothalamus from propranolol and phentolamine treatments in CSF experiment), which was unexpected.

TGFβ is generally regarded as an anti-inflammatory cytokine (Marie et al., 1996; Sanjabi et al., 2009; Zhou, Spittau & Krieglstein, 2012) and it is therefore possible that increased TGFβ expression in response to SF is a homeostatic mechanism to inhibit the action of pro-inflammatory cytokines. On the other hand, there are examples of pro-inflammatory effects from TGFβ (reviewed in Morikawa, Derynck & Miyazono, 2016). TGF β has been shown to promote the differentiation of T helper 17 cells in conjunction with IL-6, which stimulates inflammation and amplifies autoimmune conditions (Korn et al., 2009). There is also a synergistic relationship between TGFβ and IL-4 that encourages the development of T cells which produces cytokines IL-9 and IL-10. These IL-9 and IL-10 producing T cells promote tissue inflammation and do not engage in suppressive activity (Zhou et al., 2008). Further research is needed to determine the exact role that TGF β plays in regulating SF-mediated immune responses.

In the ASF experiment, phentolamine (non-selective α-adrenergic blocker) was effective at decreasing pro-inflammatory gene expression in hypothalamus of ASF and control mice, while having no effect upon pre-frontal cortex or hippocampus. The same effect was observed in EOWAT with a reduction in IL1β. In contrast, in the CSF experiment, the effect of phentolamine on cytokine gene expression was more widespread, and there was an increased occurance of interaction effects in comparison to the ASF Experiment. Among CSF mice, phentolamine decreased IL1β expression in prefrontal cortex and TNFα expression in hippocampus compared to vehicle. In addition, phentolamine reduced TNFα expression in spleen and decreased TGFβ expression in heart of CSF mice relative to vehicle. These findings suggest that mice experiencing CSF respond differently to an α-adrenergic receptor blockade than mice only experiencing 24 h of SF. Previous research has shown that increased sympathetic tone induced by chronic stressors, including chronic sleep loss, diminishes α-adrenergic receptor quantity and sensitivity in the brain and peripheral vasculature (Grote, Kraiczi & Hedner, 2000; Kim et al., 2013). In relation to the immune system, a number of immune cells are regulated by α-adrenergic receptor stimulation including cell proliferation, cytokine production, lytic activity and antibody production (Grisanti, Perez & Porter, 2011). In this study, the effect of phentolamine on inflammatory responses could involve either changes in blood flow to various target tissues or direct interactions on immune cells, although further study is warranted.

We originally predicted that propranolol (non-selective β-adrenergic blocker) would have a greater effect upon inflammatory responses than phentolamine. However, this hypothesis was not clearly supported. For example, In the CSF experiment, phentolamine treated mice exhibited decreased mRNA expression of IL1β, TNFα, and TGFβ while propranolol only decreased IL1β expression. These data suggest that these tissues are sensitive to both α- and β-adrenergic receptor blockade, and catecholamines play a role in mediating inflammation in these tissues. Our results support the hypothesis that catecholamines influence the distribution and activity of β-adrenergic receptors in the brain due to chronic sleep loss (Radulovacki & Micovic, 1982; Kim et al., 2013). However, some tissues exhibited differential responses to propranolol, which highlights the complexity of the effect that catecholamines have upon regulating inflammatory responses. To help explain these disparate effects, it has been postulated that the net effect of stimulating or inhibiting adrenergic receptors on immune cells is not straightforward, as there are a variety of factors at play that can alter the outcome, such as the activation state of the target cell, the proximity of the cell to the drug, and the pattern of expression of adrenergic receptors (Pongratz & Straub, 2014). To control these variables, it would be ideal to examine the effects of these adrenergic antagonists using cell lines or isolated cells derived from mice subjected to SF.

Serum corticosterone concentration was elevated as a result of CSF and an interaction effect of sleep and drug treatments showed that serum corticosterone in CSF mice receiving propranolol was lower than other groups. In response to ASF and CSF, the hypothalamic-pituitary-adrenal axis (HPA axis) was affected as evidenced by elevated serum corticosterone (Cort) levels. The elevation in Cort is thought to act in an anti-inflammatory manner in response to a stressor, e.g., sleep fragmentation, to suppress the action of pro-inflammatory cytokines produced by the innate immune system (reviewed in Besedovsky & Del Rey, 1996; but also see Glover et al., 2009; Lima et al., 2014). As seen in previous experiments from our lab, we show both acute and chronic sleep fragmentation resulted in elevated Cort levels (Dumaine & Ashley, 2018; Mishra et al., 2020). However, Cort concentration was lower in CSF receiving vehicle than ASF receiving vehicle, which was another finding duplicated from our lab (Mishra et al., 2020). We report female mice subjected to ASF and given phentolamine exhibited a reduced production of Cort, whereas treatment with propranolol reduced Cort concentrations in CSF mice, suggesting that α-adrenergic receptors are integral in regulating the HPA axis in ASF conditions, while β-adrenergic receptors regulate the HPA axis in response to CSF. Both α- and β-adrenergic receptors have been implicated in HPA axis regulation (Bugajski et al., 1995), and the reduction of circulating Cort concentrations has been attributed to the down regulation of SNS activity (Lowrance et al., 2016; Mishra et al., 2020). We suggest that reduction of Cort in CSF mice receiving propranolol is the product of a combined effect of an adaptive neurologic response to chronic stress, CSF, and the antagonistic action of the β-adrenergic receptor blocker, propranolol. The abundance of hypothalamic β-adrenergic receptors decreases in response to chronic stress (Stone & Platt, 1982; Thorsdottir et al., 2019), therefore increasing the efficacy of propranolol and inhibiting catecholamine binding at the hypothalamus (Tuross & Patrick, 1986), which in turn reduces adrenocorticotropic hormone (ACTH) release (Spiga & Lightman, 2015) and consequently, Cort secretion.

In the past, there has been a general hesitancy of biomedical researchers to use female mice because it was assumed that females are more variable than males and should be tested at each stage of their 4-day estrous cycle. Because we did not assess the reproductive status of female mice in our study, it cannot be ruled out that stage of estrous contributes to variation in response. However, a meta-analysis revealed that randomly cycling female mice were no more variable for a variety of physiological, behavioral, and molecular traits than male mice (Prendergast, Onishi & Zucker, 2014), suggesting that estrous cycle plays a smaller role than originally thought. Nevertheless, future studies should evaluate whether phasing of the estrous cycle affects inflammatory responses to sleep loss in female mice.

Body mass was also affected by sleep treatment, in which control mice (CON) increased body mass from Week 2 (0.65%) to Week 4 (2.61%) while CSF decreased body mass from Week 2 (−2.41%) to Week 4 (−6.53%). These findings are in contrast to male mice, where 8 weeks of CSF leads to body mass gain (Carreras et al., 2015). This sexual difference in body mass regulation in response to CSF needs to be explored further.

## Conclusions

To our knowledge, this is the first study assessing the effects of adrenergic receptor blockade upon inflammatory responses to either ASF or CSF. The changes observed in inflammatory responses appear to be representative of the activation of SNS and HPA and were correlated with SF duration. Additionally, there was a tissue-dependent response to phentolamine and propranolol, suggesting that both types of adrenergic receptors play a role in regulating inflammatory responses to SF (Tables 1 and 2). These results are also consistent with our previous findings from our lab and establishes that exposing mice to four weeks of CSF achieves comparable inflammatory effects seen in eight weeks of CSF (Mishra et al., 2020). Lastly, this study provides evidence that both α- and β-adrenergic receptors are involved in the SNS regulation of inflammatory responses to SF, but their contribution likely differs relative to acute versus chronic SF. There were twice as many significant interactions between sleep fragmentation and adrenergic function during CSF than ASF, and both adrenergic receptor types were recruited due to the prolonged exposure to SF. Therefore, future directions for this body of work should be aimed at identifying which isoform(s) of α- and β-adrenergic receptors are most influential in regulating this pro-inflammatory phenotype. Lastly, results from the research could aid in the development of therapeutics that specifically target α- and β-adrenergic receptors to mitigate inflammation in patients with OSA or other sleep disorders.

##  Supplemental Information

10.7717/peerj.11616/supp-1 Supplemental Information 1 Acute Sleep Fragmentation Experiment Real-Time PCR Data for gene IL1β Each data point indicates the mRNA expression level of the target gene.

10.7717/peerj.11616/supp-2 Supplemental Information 2 Acute Sleep Fragmentation Experiment Real-Time PCR Data for gene TNFα Each data point indicates the mRNA expression level of the target gene.

10.7717/peerj.11616/supp-3 Supplemental Information 3 Acute Sleep Fragmentation Experiment Real-Time PCR Data for gene TGFβ Each data point indicates the mRNA expression level of the target gene.

10.7717/peerj.11616/supp-4 Supplemental Information 4 Chronic Sleep Fragmentation Experiment Real-Time PCR Data for gene IL1β Each data point indicates the mRNA expression level of the target gene.

10.7717/peerj.11616/supp-5 Supplemental Information 5 Chronic Sleep Fragmentation Experiment Real-Time PCR Data for gene TNF *α* Each data point indicates the mRNA expression level of the target gene.

10.7717/peerj.11616/supp-6 Supplemental Information 6 Chronic Sleep Fragmentation Experiment Real-Time PCR Data for gene TGF *β* Each data point indicates the mRNA expression level of the target gene.

10.7717/peerj.11616/supp-7 Supplemental Information 7 Chronic Sleep Fragmentation Body Mass Data Each data point indicates the change in body mass (g) over the course of the chronic sleep fragmentation experiment.

10.7717/peerj.11616/supp-8 Supplemental Information 8 Acute and Chronic Sleep Fragmentation Experiment Corticosterone ELISA Data Each data point indicates the corticosterone concentration.

10.7717/peerj.11616/supp-9 Supplemental Information 9 The ARRIVE guidelines 2.0: author checklist

## Additional Information and Declarations

### Data Availability

The following information was supplied regarding data availability: The raw data for the Real-Time PCR results, the raw measurements for the change in body mass during the chronic sleep fragmentation experiment and the raw data for the ELISA results are available in the Supplemental Files.
